# The association of the perioperative neutrophil-to-lymphocyte ratio with wound healing in patients with Wagner grade 3 and 4 diabetic foot ulcers after tibial cortex transverse transport surgery: a prospective observational cohort study

**DOI:** 10.3389/fendo.2024.1420232

**Published:** 2024-10-29

**Authors:** Sichun Zhao, Fudie Guo, Yonghui Zang, Rong Hu, Xianjun Yu, Hongmei Zhang, Tingting Xie, Xiaoya Li, Chunxia Bai, Haoran Shi, Dingwei Zhang

**Affiliations:** Orthopaedics, Mianyang Central Hospital, School of Medicine, University of Electronic Science and Technology of China, Mianyang, China

**Keywords:** diabetic foot ulcer, Wagner grade 3 and 4, tibial cortex transverse transport technique, neutrophil-to-lymphocyte ratio (NLR), wound healing

## Abstract

**Background:**

Diabetic foot ulcers (DFUs) are severe complications of diabetes, involving multiple etiological factors including neuropathy, vascular insufficiency, and impaired wound healing. The global burden of DFUs is substantial, with significant morbidity and high management costs. Recent advancements have introduced the tibial cortex transverse transport (TTT) technique, which has shown promising results in the management of severe DFUs by promoting angiogenesis and immunomodulation.

**Methods:**

This prospective cohort study enrolled patients with Wagner grade 3 and 4 DFUs, treated using the TTT technique from May 2022 to September 2023 at MianYang Central Hospital. The study assessed the influence of TTT on the perioperative neutrophil-to-lymphocyte ratio (NLR), an established biomarker of systemic inflammation, and its correlation with wound healing outcomes.

**Results:**

A total of 82 patients were enrolled, with 66 completing the study. The survival analysis revealed that patients with a lower preoperative NLR experienced significantly faster wound healing compared with a high NLR (log rank test *P*< 0.05; hazard ratio (HR) = 0.46; 95% CI: 0.26–0.83). The optimal NLR cutoff values (4.25) were established to predict wound healing times for DFUs. The median NLR was significantly different before TTT surgery, 3 days after TTT surgery, and 1 month after TTT surgery (*P*<0.05).

**Conclusion:**

The TTT technique significantly influences the perioperative NLR and is associated with improved wound healing in DFU patients. The perioperative NLR serves as an effective predictive biomarker for wound healing outcomes, highlighting the significance of interventions targeting NLR values in perioperative management strategies and postoperative monitoring protocols for the treatment of diabetic foot ulcers (DFUs) in clinical practice.

## Introduction

Diabetic foot ulcers (DFUs) represent a significant and challenging complication of diabetes mellitus, characterized by chronic wounds that develop on the lower extremities. These ulcers emerge from a complex interplay of factors, including neuropathy, vascular insufficiency, and impaired wound healing, all exacerbated by the metabolic abnormalities associated with diabetes. Globally, DFUs afflict millions of individuals each year, with approximately 18.6 million cases reported worldwide and 1.6 million in the United States alone annually (1). The management of DFUs presents a formidable clinical obstacle, often necessitating a multidisciplinary approach involving podiatrists, endocrinologists, vascular surgeons, and wound care specialists. Despite advancements in treatment modalities such as wound debridement, offloading techniques, and advanced wound dressings, DFUs remain stubbornly resistant to healing and are associated with considerable morbidity and mortality (2). In previous studies, the authors developed a new technique named tibial cortex transverse transport (TTT) and applied it in the treatment of severe and recalcitrant DFUs (3–5). The TTT procedure involves the creation of a corticotomy or osteotomy in the tibial cortex, followed by the gradual transverse movement of the bone segments using an external fixation device. By inducing controlled mechanical stress on the bone, TTT stimulates bone regeneration and angiogenesis, promoting tissue healing and ulcer closure (6). The results showed a higher wound healing rate, greater extremity salvage, and a lower recurrence rate in the TTT treatment group than in the other treatment groups (3–5, 7, 8). Postoperative radiographic studies also revealed a significant increase in neovascularization and perfusion in the treated extremities (3–5). Treatment with TTT in patients with severe diabetic foot conditions associated with systemic inflammatory response syndrome (SIRS) has shown promising outcomes in terms of ameliorating systemic inflammation and accelerating ulcer healing (7). In a rat model, TTT accelerated wound healing via enhanced angiogenesis and immunomodulation (9). This observation led to the proposition that one of the potential mechanisms underlying the efficacy of TTT in promoting the healing of DFUs lies in its ability to modulate the inflammatory response, thereby facilitating immunomodulation.

The NLR has emerged as a pivotal biomarker to discern the delicate equilibrium between inhibitory and excitatory facets within the immune system (10). Neutrophils, key effectors of the innate immune response, exhibit a proclivity to traverse the vascular wall, unleashing a cascade of inflammatory mediators, including superoxide radicals, cytokines, and an array of proteolytic enzymes. This orchestrated release significantly contributes to endothelial damage, thereby exacerbating vascular dysfunction. Conversely, lymphocytes play a crucial role in modulating the impact of neutrophils and exerting an anti-atherosclerotic influence. Their regulatory functions serve to counterbalance the proinflammatory actions of neutrophils, thereby mitigating the adverse consequences of endothelial injury. The significance of the NLR lies in its ability to reflect the dynamic interplay between these cellular components of the immune system. A high NLR indicates an imbalance skewed toward increased neutrophilic activity, correlating with elevated endothelial damage and dysfunction (11). Consequently, high NLRs serve as a prognostic indicator for adverse outcomes in various pathological contexts. The neutrophil-to-lymphocyte ratio (NLR) is derived from the quotient of the neutrophil count to the lymphocyte count obtained through a standard differential blood cell count test. This metric presents a cost-effective and readily accessible means of gauging immune system activity, especially when juxtaposed with more specialized markers such as matrix metalloproteinases or growth factors. Some studies have shown that the NLR is a predictor of DFUs during treatment, and an elevated NLR was associated with bad outcome in patients with DFUs (12–19). However, no studies have focused on the association between the perioperative NLR and diabetic foot wound outcomes following TTT surgery.

Thus, we selected the NLR as a biomarker to assess systemic inflammation and the immunomodulatory response in diabetic foot patients following TTT surgery. The aims of this study were to investigate whether the TTT technique influences the NLR and thus the ability of the immune system to relieve systemic and local inflammation and promote diabetic foot wound healing and to determine the predictive value of the perioperative NLR for wound healing in Wagner grade 3 and 4 diabetic foot patients following TTT surgery.

## Materials and methods

In this prospective cohort clinical study, we consecutively treated patients with DFUs using the TTT technique between May 13, 2022 and September 2023 in the MianYang Central Hospital Department of Orthopaedics. The study protocol was approved by the Biomedical Ethics Committee of MianYang Central Hospital and was conducted according to the principles of the Declaration of Helsinki prior to enrollment. Written informed consent was obtained from each patient prior to enrollment.

### Inclusion criteria

Following are the inclusion criteria: (1) at least 18 years of age, (2) a diagnosis of diabetes foot according to the American Diabetes Association criteria (20), (3) a lack of response to preoperative treatment (e.g., debridement) for at least 3 months, (4) Wagner grade 3 or 4 DFUs, (5) ability to undergo continuous follow-up and be hospitalized for 1 month, and (6) the superficial femoral artery and popliteal artery were not severely stenotic or occluded (≥80% of the lumen as evaluated by CTA and ultrasound), and at least one branch of the anterior tibial artery, posterior tibial artery, or fibular artery was unobstructed at the ankle joint plane in Doppler ultrasound.

### Exclusion criteria

Following are the exclusion criteria: (1) a malignant disease in the ulcers, (2) ulcers extending above the ankle but too close to the surgical area (i.e., the distance between the ulcer margin and the surgical area was less than 5 cm), (3) ulcers without a diagnosis of diabetes or active Charcot arthropathy, (4) infection of the calf in the surgical area, (5) acute critical limb ischemia, (6) autoimmune diseases, (7) current use of corticosteroids, immunosuppressive drugs, and/or chemotherapy, (8) end-stage renal disease, (9) a history of myocardial infarction or/and stroke within 3 months of the study, and (10) femoral–popliteal artery severe stenosis [≥80% of the lumen as evaluated by computed tomography angiography (CTA) and ultrasound] or occlusion.

### Clinical evaluation and laboratory tests

The ulcer Wagner classification, ankle-brachial index (ABI) in the resting state, history of diabetes, fasting blood glucose, postprandial blood glucose, well-controlled blood glucose, and demographic data, such as age and sex, were evaluated according to the Chinese Association of Orthopaedic Surgeons (CAOS), Taskforce Group of Tibial Cortex Transverse Transport Technique for the Treatment of Diabetic Foot Ulcers (21).

Blood cell counts, including neutrophil counts and lymphocyte counts, were performed on samples collected from each participant, who were in a fasted state, from the median cubital vein in the ward on admission before TTT surgery, 3 days after TTT surgery, and 1 month after TTT surgery. In order to detect the systemic inflammation and the immunomodulatory response effect of TTT surgery itself, we collected 3-day postoperative blood cell counts to avoid the influence of factors such as anesthesia, wound debridement, and other confusing surgery-related factors. We collected blood samples from the participants at 1 month after TTT surgery for the reason that each patient received the same medical procedure during hospitalization and completed the whole TTT procedure. Data using other laboratory test indicators, such as hemoglobin, hemoglobin A1c, albumin, and the lymphocyte-to-monocyte ratio (LMR), were also collected. All the data were collected and recorded by an independent observer who did not participate in the treatment program.

### TTT technique and treatment

Under general anesthesia, spinal anesthesia, or femoral nerve blockage, a rectangular cortex (6 cm * 2 cm) was osteotomized from the anteromedial aspect of the proximal tibia using a minimally invasive osteotomy device and attached to a monolateral external fixator as described previously (3). Following TTT, aggressive surgical debridement was performed to remove necrotic and/or infected tissues, drain abscesses, and open fistulas based on international guidelines (22). The infected bone was removed, and minor amputations (resections through or distal to the ankle) were performed when indicated. Deep tissue samples were collected for histopathological and microbiological examination to identify causative organisms and their antibiotic sensitivities.

### Aftercare and cortex transport

After a 7-day latency period, the distraction of the osteotomized cortex was initiated at a rate of 1 mm per day, divided into six sessions. The distraction period was 2 weeks—1 week medially pull, followed by 1 week laterally push. Aftercare included daily dressing changes and pin site care. Early on the 2nd day after surgery, the patients were allowed to place partial weight on the operated limbs using crutches. When the distraction was complete, the external fixator was removed. At 4 weeks later, the patients were allowed to ambulate bearing their full weight.

### Management protocol

Throughout the treatment process, we focus on regulating blood glucose levels, correcting hypoproteinemia and electrolyte disturbances, maintaining renal function, and administering medications to improve microcirculation. In patients with local wound infection and osteomyelitis without systemic infection symptoms, TTT + debridement was applied for treatment. Antibiotics were used only when the patients experienced systemic symptoms of infection during the study. Systemic symptoms of infection means two or more signs of systemic inflammatory response syndrome: (1) temperature >38°C or<36°C, (2) heart rate >90/min, (3) respiratory rate >20/min or PaCO_2_<32 mmHg, and (3) white blood cell count >12,000/uL or<4,000/μL or 10% immature forms. Wound dressing changes were conducted by two wound specialists who were blinded to the wound status before the operation. These wound specialists adopted a unified dressing change process involving wound drainage, limited clearance of infected tissues, and dressing exchange in their clinical practice. No other additional wound healing therapies, such as negative pressure wound therapy or antimicrobial dressings, were used. The patients received dressing changes daily during the first month in our ward and approximately three times a week when they went home. When the wound deteriorated, such as aggravated infection and necrosis, a wound specialist who did not participate in the study made the decision to continue or cease wound dressing and therefore to proceed with amputation, debridement surgery, skin flap surgery, or any other treatment. The wound healing time was recorded by an independent observer who was blinded to all study conditions.

### Outcomes

The primary outcome was the association between the NLR and wound healing time. Wound healing was defined as complete epithelialization and no need for dressing changes during the study. Patients who died or underwent amputation during the study period were censored. Thus, patients were grouped by wound healing or not. Wound healing time was calculated from the day after TTT surgery. The second outcome was the difference in the NLR between baseline, 3 days after TTT surgery, and 1 month post-TTT surgery.

### Statistical analysis

Continuous variables are expressed as the means (standard deviations, SDs) or medians (interquartile ranges, IQRs). The normality of the distribution of the data was assessed by using the Kolmogorov−Smirnov test. Normally distributed numeric variables are expressed as mean ± SD, while nonnormally distributed variables are expressed as the median (Q1, Q3). Categorical variables are expressed as *n* (%). Differences in the NLR between baseline and 1 month post-TTT surgery were compared with paired-sample Wilcoxon signed rank tests. Statistical significance was set at an α level<0.05 for two-sided comparisons.

The R language 4.2 software was used to create receiver operating characteristic (ROC) curves to test the overall discriminative ability of the NLR for wound healing time. The Youden index was calculated as sensitivity + specificity - 1. A two-tailed value of *P*< 0.05 was considered significant. The optimal cutoff value was determined by the Youden index. Low and high NLRs were classified by the optimal cutoff value. Kaplan–Meier survival analysis and log rank tests were used to compare wound healing times between the low- and high-NLR groups. Variables that were considered clinically relevant and checked with directed acyclic graph were entered into a multivariate Cox proportional-hazards regression model. Variables for inclusion were carefully chosen, given the number of events available, to ensure parsimony of the final model. A value of *p<*0.05 was considered to indicate statistical significance. Unstandardized coefficients are represented as β ± (standard error, SE), and the hazard ratios (HRs) with 95% CIs were calculated. According to the sample size formula of one single population rate, *n* = $(\frac{\mu }{\Delta }{)}^{2}$ × *p* (1 — *p*) (*p* means population rate, *p* = 20%, △ means allowable error, △ = 0.1, and *μ* = 1.96), we calculated the minimum sample size to be 62.

## Results

**Figure 1:**
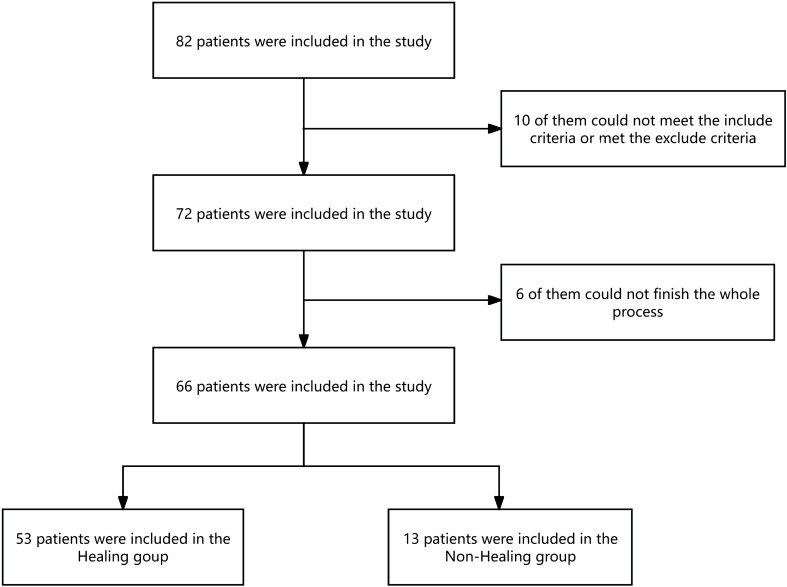
Flow diagram of participants.

In total, 82 patients were enrolled in the study, 66 of whom completed the entire study (**Figure 1**). The median age was 60.50 years old, with 47 male and 19 female patients of the total cohort. At the end of the follow-up period, 53 patients were included in the wound healing group, and 13 patients were included in the nonhealing group. The wound healing rate is 80.3%. The median time to complete wound healing was 75.06 days. In the nonhealing group, eight patients died, and five patients underwent amputation during the study. The wounds of the other 53 patients all healed. One patient experienced an incision infection, and one patient experienced a nail tract infection during the follow-up period; the wounds of both of these patients healed with wound dressing changes (**Figure 1**). The baseline data of the patients in the healing group and the nonhealing group are shown in **Table 1**. We found that, compared with patients in the wound healing group, patients in the nonhealing group were older, had a greater NLR, had more Wagner grade 4 disease, had a lower glomerular filtration rate (GFR), had a lower ABI, had a lower LMR, and had more diabetic eye disease (statistically significant at *P*<0.05) (**Table 1**).

**Table 1:** Preoperative variables of the total cohort, healing group, and non-healing group.

Variables	Total (*N* = 66)	Healing group (*N* = 53)	Non-healing group (*N* = 13)	Diff (95%CI)		*p*
Age (years)	60.50 (54.25, 74.25)	60.00 (53.00, 72.00)	72.00 (60.00, 76.00)	-7.00 (-16.00, -1.00)	*z*: 2.074	0.038*
Wagner classification, *n* (%)					*z*: 3.409	<0.001*
3	38 (57.6%)	36 (67.9%)	2 (15.4%)			
4	28 (42.4%)	17 (32.1%)	11 (84.6%)			
History of diabetes (years)	10.00 (5.00, 16.00)	10.00 (3.00, 16.00)	10.00 (10.00, 20.00)	-4.00 (-10.00, 0.00)	*z*: 1.754	0.079
GFR (ml/min)	56.90 (43.38, 72.40)	59.70 (47.40, 74.30)	43.90 (39.90, 55.00)	16.80 (4.00, 28.90)	*z*: 2.556	0.011*
Hemoglobin A1c (%)	9.30 (8.33, 10.97)	9.70 (8.60, 11.30)	8.90 (8.10, 10.80)	0.70 (-0.70, 2.00)	*z*: 1.129	0.259
Fasting blood glucose (mmol/L)	9.82 (6.42, 16.60)	9.97 (6.80, 16.59)	8.70 (5.97, 16.67)	0.69 (-2.53, 4.43)	*z*: 0.588	0.556
Postprandial blood glucose (mmol/L)	15.30 (11.20, 23.10)	16.10 (11.20, 23.10)	13.40 (11.20, 23.10)	0.50 (-2.20, 4.80)	*z*: 0.468	0.640
Hemoglobin (g/L)	103.00 (94.00, 121.00)	105.00 (94.00, 122.00)	102.00 (91.00, 106.00)	4.00 (-6.00, 17.00)	*z*: 0.790	0.429
Albumin (g/L)	33.05 (29.59, 37.50)	32.70 (30.02, 37.08)	35.26 (29.05, 37.86)	-0.12 (-3.99, 4.49)	*z*: 0.064	0.949
Total protein (g/L)	65.43 (59.18, 69.87)	65.31 (60.20, 69.27)	66.25 (58.56, 71.59)	-0.27 (-6.05, 5.63)	*z*: 0.073	0.942
ABI	1.00 (0.80, 1.10)	1.00 (0.90, 1.10)	0.80 (0.77, 1.00)	0.20 (0.00, 0.30)	*z*: 2.125	0.034*
NLR	4.15 (2.49, 5.96)	3.55 (2.27, 5.62)	5.11 (4.73, 7.16)	-1.91 (-3.12, -0.56)	*z*: 2.507	0.012*
LMR	2.65 (1.66, 3.55)	2.76 (1.92, 3.81)	1.62 (1.13, 2.14)	1.09 (0.31, 1.89)	*z*: 2.797	0.005*
Sex, *n* (%)					*χ* ^2^: 0.721	0.396
Male	47 (71.2%)	36 (67.9%)	11 (84.6%)			
Female	19 (28.8%)	17 (32.1%)	2 (15.4%)			
Well-controlled blood glucose, *n* (%)					*χ* ^2^: 1.443	0.230
No	47 (71.2%)	40 (75.5%)	7 (53.8%)			
Yes	19 (28.8%)	13 (24.5%)	6 (46.2%)			
Diabetic eye disease, *n* (%)					*χ* ^2^: 3.874	0.049*
No	23 (34.8%)	22 (41.5%)	1 (7.7%)			
Yes	43 (65.2%)	31 (58.5%)	12 (92.3%)			
Cardiovascular disease, *n* (%)					*χ* ^2^: 8.461	0.004*
No	34 (51.5%)	32 (60.4%)	2 (15.4%)			
Yes	32 (48.5%)	21 (39.6%)	11 (84.6%)			
Antibiotics, *n* (%)					*χ* ^2^: 1.301	0.254
Never use	42 (63.6%)	36 (67.9%)	6 (46.2%)			
Ever use	24 (36.4%)	17 (32.1%)	7 (53.8%)			

Data are presented as median with IQR for continuous variables and as number for categorical variables. The *P*-value is calculated by using Mann–Whitney U-test for continuous variables and Fisher’s exact test for categorical variables.

GFR, glomerular filtration rate; ABI, ankle-brachial index; NLR, neutrophil-to-lymphocyte ratio; LMR, lymphocyte-to-monocyte ratio.

^*^ *P*-value<0.05.

### Relationship between the perioperative NLR and the wound healing time

**Figure 2:**
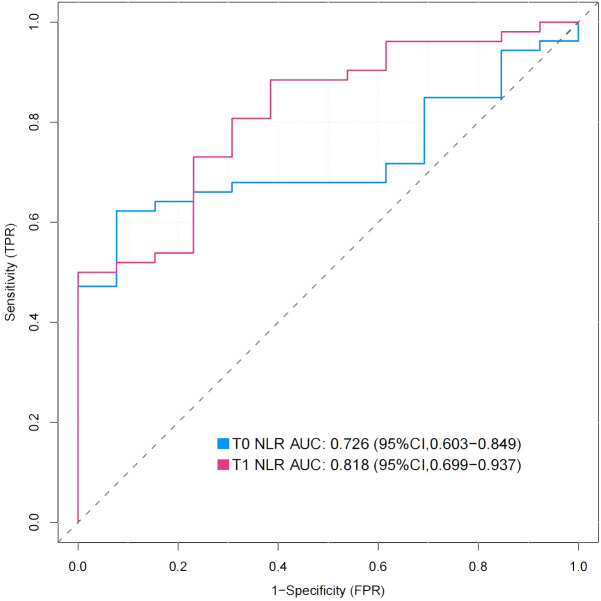
Receiver-operating characteristic (ROC) curves for preoperative NLR and 3-day postoperative NLR. The Figure showed the ROC curves for preoperative NLR(T0) and 3-day postoperative NLR(T1), whose area under the curve (AUC) were 0.726 and 0.818 respectively.

The preoperative NLR in the nonhealing group was significantly greater than that in the healing group (**Table 1**). To evaluate the predictive value of the preoperative NLR for wound healing, ROC curve analysis was performed (23). The cutoff value [4.25; specificity (spe): 92.3, sensitivity (sen): 62.3] of the preoperative NLR was determined by the Youden index, and the area under the ROC curve (AUC) was 0.726 (*P*< 0.001, 95% CI: 0.602–0.828). To evaluate the predictive value of the 3-day postoperative NLR for wound healing, ROC curve analysis was also performed. The cutoff value (6.93; spe: 77.8, sen: 72.7) for the 3-day postoperative NLR was determined by the Youden index, and the AUC was 0.811 (*P*< 0.001, 95% CI: 0.679–0.905). There was no significant difference between the preoperative NLR and the postoperative NLR (*P* > 0.05, 95% CI: -0.0437–0.236) (**Figure 2**). These data show that preoperative NLR and 3-day postoperative NLR could be a predictive biomarker for DFUs with relatively high sensitivity and specificity in patients following TTT surgery.

**Figure 3:**
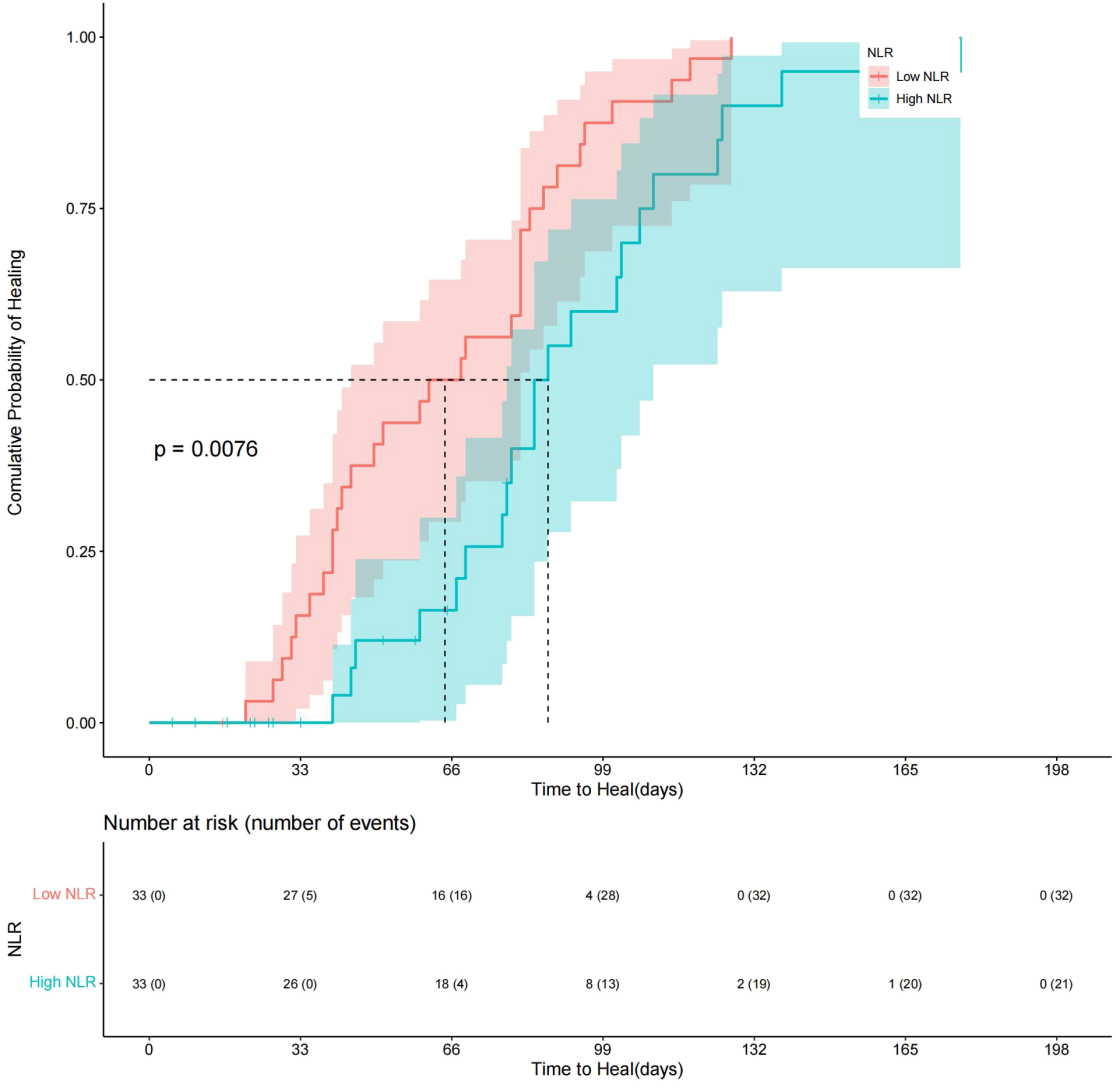
Kaplan−Meier plot comparing wound healing time between Low-NLR group and High-NLR group.About 85% of DFUs had completely healed at 14 weeks, while 97% of diabetic foot wounds had completely healed at 19 weeks in the low-NLR group after the TTT surgery.

The patients were divided into a high-NLR group and a low-NLR group according to a cutoff value of preoperative NLR (4.25). Survival distributions (time to achieve complete healing) between the high-NLR group and the low-NLR group were significantly different until the end of the study (log rank test *P*< 0.05; hazard ratio (HR) = 0.46; 95% CI: 0.26–0.83). A Kaplan−Meier plot of the low- and high-NLR groups is shown in **Figure 3**. About 85% of DFUs had completely healed at 14 weeks, while 97% of diabetic foot wounds had completely healed at 19 weeks in the low-NLR group after the TTT surgery (**Figure 3**). Variables that were considered clinically relevant and checked with directed acyclic graph were entered into a multivariate Cox proportional-hazards regression model. The preoperative GFR, preoperative hemoglobin A1c, Wagner classification, and preoperative NLR were determined as potential factors of a systemic inflammatory response for wound healing. Therefore, a multivariate Cox regression model adjusted for the preoperative GFR, preoperative hemoglobin A1c level, and Wagner classification was applied to determine the association between the high-NLR group and the low-NLR group with wound healing (a*P*< 0.05; aHR = 0.46; 95% CI: 0.23–0.91) (**Table 2**). These data indicate that a lower preoperative NLR is a strong predictor for Wagner 3 and 4 classification DFU patients to undergo TTT surgery, based on the optimal cutoff value of 4.25, and is associated with faster wound healing.

**Table 2:** Difference of potential variables between preoperation and postoperation.

Variables	Preoperation (*n* = 66)	Postoperation (*n* = 66)	Diff (95%CI)	*z*	*P*
GFR (ml/min)	56.90 (43.38, 72.40)	54.20 (39.60, 67.60)	2.55 (-0.05, 4.90)	1.919	0.055
Hemoglobin A1c (%)	9.30 (8.33, 10.97)	7.30 (6.50, 8.30)	2.20 (1.70, 2.70)	5.737	<0.001*
Fasting blood glucose (mmol/L)	9.82 (6.42, 16.60)	8.60 (6.93, 10.29)	2.73 (0.33, 4.62)	2.268	0.023*
Postprandial blood glucose (mmol/L)	15.30 (11.20, 23.10)	12.10 (9.90, 15.10)	4.45 (1.95, 6.70)	3.476	<0.001*
Hemoglobin (g/L)	103.00 (94.00, 121.00)	106.00 (97.00, 121.00)	-0.00 (-5.50, 5.50)	0.044	0.967
Albumin (g/L)	33.05 (29.59, 37.50)	34.83 (31.30, 39.50)	-1.27 (-2.88, 0.55)	1.371	0.173
Total protein (g/L)	65.43 (59.18, 69.87)	67.14 (61.16, 71.95)	-1.47 (-3.33, 0.69)	1.426	0.156
ABI	1.00 (0.80, 1.10)	1.00 (0.90, 1.10)	0.00 (-0.10, 0.05)	0.413	0.684
NLR	4.15 (2.49, 5.96)	3.17 (2.45, 4.66)	0.97 (0.15, 1.81)	2.260	0.023*
LMR	2.65 (1.66, 3.55)	2.95 (2.16, 3.70)	-0.28 (-0.71, 0.22)	1.158	0.251

Data are presented as median with IQR for continuous variables and as number for categorical variables. The *P*-value is calculated by using Wilcoxon signed rank tests.

GFR, glomerular filtration rate; ABI, ankle-brachial index; NLR, neutrophil-to-lymphocyte ratio; LMR, lymphocyte-to-monocyte ratio.

^*^ *P*-value<0.05.

### Preoperative, 3-day postoperative, and 1-month postoperative differences in the NLR

**Figure 4:**
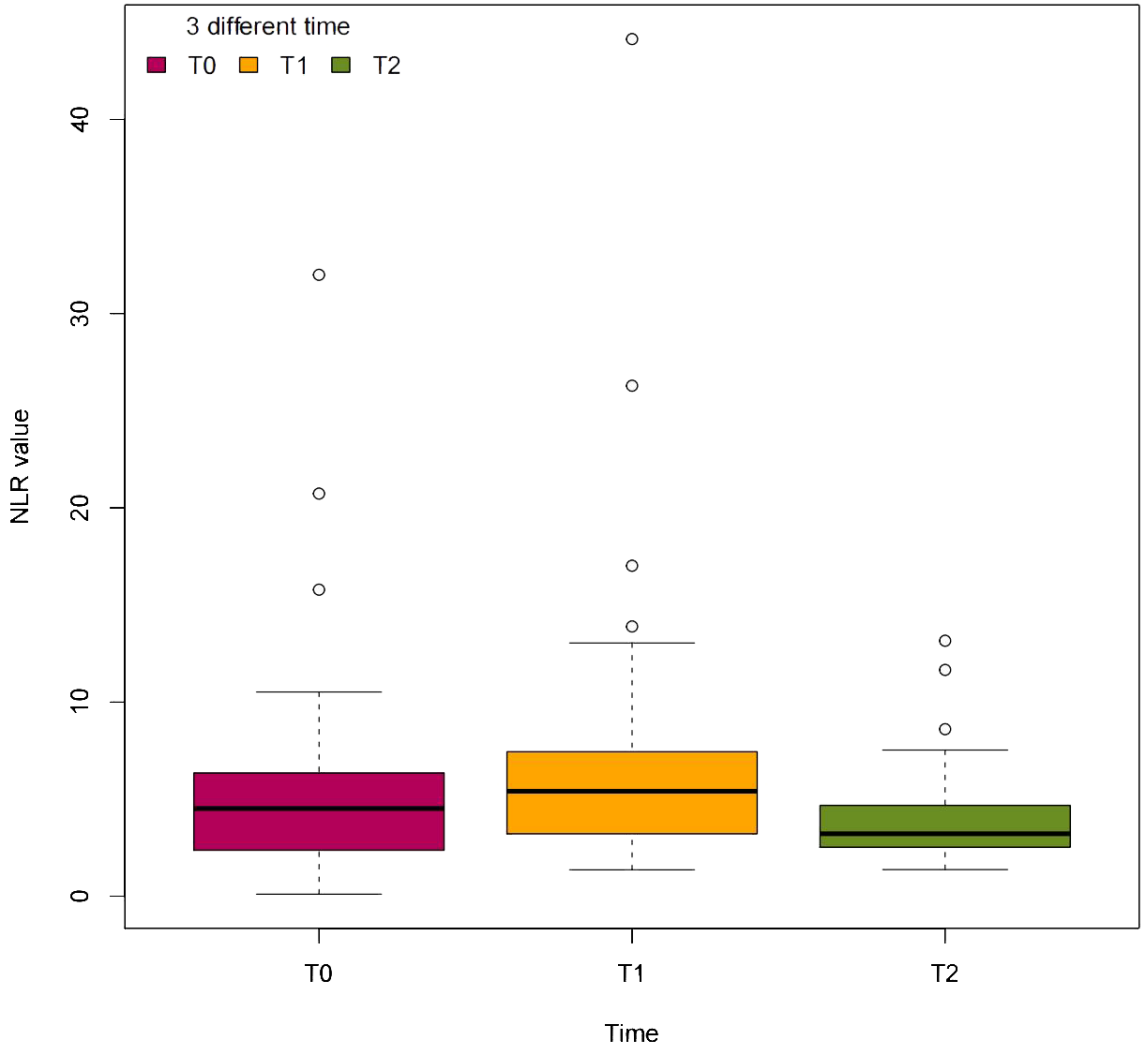
The dynamic change of NLR value at 3 different time. T0=before TTT surgery, T1=3 days after TTT surgery, T2=1 month after TTT surgery.

The preoperative hemoglobin A1c, fasting blood glucose, and postprandial blood glucose levels were significantly greater than the postoperative levels. However, the GFR, hemoglobin, albumin, total protein, ABI, and LMR were not significantly different before and 1 month after TTT surgery (**Table 3**). The median NLR was significantly different before TTT surgery (T0), 3 days after TTT surgery (T1), and 1 month after TTT surgery (T2) (*P*<0.05) (**Figure 4**). These data show that TTT surgery triggers an immune inflammatory response, which is initially accompanied by an increased NLR value. This value then decreases during the pulling and pushing of the bone cortex, helping to sustain the anti-inflammatory effect and promote wound healing.

**Table 3:** Univariate and multivariate Cox proportional-hazards regression model for the variables.

Variables	Beta	S.E.	*Z*	*P*	HR (95%CI)	m_Beta	m_S.E.	m_Z	aP	aHR (95%CI)
GFR	-0.01	0.01	-0.99	0.325	0.99 (0.98–1.01)	-0.66	0.36	-1.84	0.066	0.52 (0.25–1.04)
Hemoglobin A1c	-0.09	0.06	-1.53	0.127	0.91 (0.81–1.03)	-0.01	0.01	-1.45	0.147	0.99 (0.97–1.00)
Wagner classification										
3					Ref					Ref
4	-1.04	0.33	-3.16	0.002*	0.35 (0.18–0.67)	-0.06	0.07	-0.84	0.403	0.95 (0.83–1.08)
NLR group										
Low NLR					Ref					Ref
High NLR	-0.77	0.29	-2.62	0.009*	0.46 (0.26–0.83)	-0.78	0.35	-2.22	0.027*	0.46 (0.23–0.91)

GFR, glomerular filtration rate; HR, hazard ratio; CI, confidence interval.

^*^ *P*-value<0.05.

## Discussion

The aims of this study were to investigate whether the TTT technique influences the NLR and thus the ability of the immune system to relieve systemic and local inflammation and promote diabetic foot wound healing and to determine the predictive value of the perioperative NLR for wound healing in Wagner grade 3 and 4 diabetic foot patients following TTT surgery. We found that preoperative NLR and 3-day postoperative NLR could be a predictive biomarker for DFUs with relatively high sensitivity and specificity in patients following TTT surgery. A low preoperative NLR is a good indication for Wagner 3 and 4 classification DFU patients to receive TTT surgery. TTT surgery is a trigger to motivate immune inflammatory response and then decreases it during the pulling and pushing of the bone cortex to maintain the anti-inflammatory effect to promote wound healing.

Complete healing of diabetic foot wounds is difficult due to infection, peripheral artery disease, diabetic neuropathy, and other reasons (24). Previous studies have shown that the TTT technique can promote wound healing in diabetic patients by increasing angiogenesis in the limb via. However, Liao and colleagues reported that, in the early stage after TTT, the blood flow volume and velocity and the plantar microcirculation perfusion of the affected limb of diabetic foot patients with nonarterial stenosis decreased compared with those at baseline, while those of diabetic foot patients with arterial stenosis increased significantly compared with those at baseline, although both groups of patients had smoothly healed DFUs (25). Our study did not show that the TTT procedure could increase the blood supply according to the ABI, which is consistent with the findings of Liao’s study. Our patients might have good blood supply with relatively high ABI values compared with those in previous studies. However, the ABI has low sensitivity and specificity in predicting diabetic wound healing, with low prognostic accuracy for ulcer healing in the clinic (26).

Thus, angiogenesis might not be the main reason for diabetic wound healing following TTT treatment in patients with a relatively good blood supply. We found that under the premise of the same local wound management procedure, treatment of diabetic foot wounds with the TTT technique can reduce tissue redness and swelling and wound exudation that accompanies wound healing. We used antibiotics only when the patients experienced systemic symptoms of infection during the study. This finding might indicate that TTT can reduce local and systemic inflammatory reactions. One previous study focused on patients classified as having Wagner grade 3 or higher DFUs with SIRS (7). Postoperative inflammatory indicators such as white blood cell (WBC) count, C-reactive protein (CRP), and erythrocyte sedimentation rate (ESR) were significantly lower during wound healing than before surgery, which showed that TTT significantly stimulated immune cells, thereby playing a crucial role in diabetic foot wound healing. Studies have demonstrated that TTT can facilitate the transition of M1 macrophages into M2 macrophages during the proliferation phase of wound healing. This process promotes the polarization balance of macrophages and the reconstruction of anti-inflammatory functions, ultimately accelerating wound healing (27–29).

The determination of whether the NLR can be used as a biomarker holds immense promise for unraveling the complexities of immune-mediated vascular pathologies. The utility of the NLR as a biomarker extends beyond mere prognostication to informing therapeutic interventions aimed at restoring immune homeostasis and ameliorating vascular dysfunction. Remarkably, despite the osteotomy site being in the tibial diaphysis, the observed healing of foot ulcers suggests that a systemic response is elicited by TTT. Our study revealed that, compared with before TTT surgery, the NLR increased 3 days after TTT surgery and decreased during pulling and pushing of the bone cortex until 1 month after the operation. This systemic reaction may be attributed to osteotomy mobilizing the immune inflammatory response, which first increases the NLR and then decreases it during the pulling and pushing of the bone cortex to maintain the anti-inflammatory effect to promote wound healing. We found that the NLR value significantly decreased throughout the entire TTT procedure, coinciding with wound healing. This indicates the significance of interventions targeting NLR values in perioperative management strategies and postoperative monitoring protocols for the treatment of diabetic foot ulcers (DFUs) in clinical practice.

The Wound, Ischaemia, and Foot Infection (WIfI) Classification of Limb Threat has been shown to predict wound healing in DFUs (30). A higher score on the WIfI scale is associated with lower extremity amputation and morbidity and can be used to determine the need for revascularization. WIfI scores of 1, 2, 3, and 4 were associated with 1-year amputation rates of 0%, 8%, 11%, and 38%, respectively. However, it is sometimes difficult to accurately and simply evaluate wound infection and peripheral artery disease in the clinic (30, 31). Sometimes wound culture, a positive probe-to-bone test, plain film radiography, magnetic resonance imaging, and even bone biopsy are needed to assess the extent of infection. Some hospitals do not have the capabilities to perform ABI, toe systolic blood pressure index, transcutaneous oximetry, or skin perfusion pressure examinations to evaluate peripheral artery disease.

The NLR is a cost-effective and readily accessible biomarker to gauge immune system activity. Chen et al. demonstrated that a higher NLR may serve as a reliable predictive biomarker for mortality in patients undergoing DFU-related amputations (32). Similarly, Demirdal et al. found that NLR levels were higher in patients who underwent amputations compared to those who did not, suggesting that NLR could be utilized to predict the risk of DFU-related amputations (14). The studies have focused on different endpoints. Our focus was on the wound healing rate and time because it can be challenging for clinical doctors to make the best clinical decisions for patients with Wagner grade 3 and 4 DFUs to receive TTT technique. The cutoff value of the preoperative NLR (4.25) used in our study to predict diabetic foot wound healing was similar to that (4.19) used in previous studies of vascular surgery for the same purpose (13). We found that patients in the low-NLR group were more likely to experience complete wound healing according the cutoff value. Lower preoperative NLR levels correlate with faster wound healing (hazard ratio (HR) = 0.46; 95% CI: 0.26–0.83). The US Wound Registry of 71,957 diabetic foot ulcers showed healing of approximately 30%–40% at 12 weeks, and 23% of the ulcers were still unhealed at 12 months. We found that 85% of diabetic foot wounds had completely healed at 14 weeks, while 97% of diabetic foot wounds had completely healed at 19 weeks in the low-NLR group after the TTT technique (33).

Our study has several limitations. First, we acquired the ABI via a blood pressure cuff connected with a monitor, which might have confounded the real values. Thus, we could not determine the real effect of TTT on angiogenesis. Second, diabetic foot wound healing is a long and complicated process even following TTT surgery. Although we used the same procedure to treat the patients for strictly potential confounding factors during the first month of hospitalization, we cannot strictly manage the patient’s condition after discharge, such as mental and nutritional status, which might have influenced the outcomes. Third, there were inherent limitations to the observational study that we cannot avoid, such as potential confounding factors and selection bias. Fourthly, there were limitations inherent to the use of NLR as a biomarker of systemic inflammation. Further investigations, such as exploring additional biomarkers of inflammation or conducting randomized controlled trials, are necessary to validate the observed associations.

## Conclusion

The TTT technique significantly influences the perioperative NLR and is associated with improved wound healing in Wagner grade 3 and 4 DFU patients. The perioperative NLR serves as an effective predictive biomarker for wound healing outcomes, highlighting the significance of interventions targeting NLR values in perioperative management strategies and postoperative monitoring protocols for the treatment of diabetic foot ulcers in clinical practice.

## Data Availability

All data supporting the findings of this study were available from the corresponding author on request.

## References

[1] Zhang Y Lazzarini PA McPhail SM van Netten JJ Armstrong DG Pacella RE. Global disability burdens of diabetes-related lower-extremity complications in 1990 and 2016. Diabetes Care. (2020) 43:964–74. doi: 10.2337/dc19-1614 32139380

[2] Armstrong DG Swerdlow MA Armstrong AA Conte MS Padula WV Bus SA. Five year mortality and direct costs of care for people with diabetic foot complications are comparable to cancer. J Foot Ankle Res. (2020) 13:16. doi: 10.1186/s13047-020-00383-2 32209136 PMC7092527

[3] Chen Y Kuang X Zhou J Zhen P Zeng Z Lin Z. Proximal tibial cortex transverse distraction facilitating healing and limb salvage in severe and recalcitrant diabetic foot ulcers. Clin Orthop Relat Res. (2020) 478:836–51. doi: 10.1097/CORR.0000000000001075 PMC7282570 31794478

[4] Chen Y Ding X Zhu Y Jia Z Qi Y Chen M. Effect of tibial cortex transverse transport in patients with recalcitrant diabetic foot ulcers: A prospective multicenter cohort study. J Orthop Translat. (2022) 36:194–204. doi: 10.1016/j.jot.2022.09.002 36263383 PMC9576490

[5] Qin W Nie X Su H Ding Y He L Liu K. Efficacy and safety of unilateral tibial cortex transverse transport on bilateral diabetic foot ulcers: A propensity score matching study. J Orthop Translat. (2023) 42:137–46. doi: 10.1016/j.jot.2023.08.002 PMC10509564 37736148

[6] Ilizarov GA. The tension-stress effect on the genesis and growth of tissues: Part II. The influence of the rate and frequency of distraction. Clin Orthop Relat Res. (1989) 239):263–85. 2912628

[7] Zhen P Chen Y Gao W Lin Z Zhong Z Teng Z. The effectiveness of Ilizarov technique-based transverse tibial bone transport on treatment of severe diabetic foots complicated with systemic inflammation response syndrome. Zhongguo Xiu Fu Chong Jian Wai Ke Za Zhi. (2018) 32:1261–6. doi: 10.7507/1002-1892.201805024 PMC8414156 30600665

[8] Liu J Yao X Xu Z Wu Y Pei F Zhang L. Modified tibial cortex transverse transport for diabetic foot ulcers with Wagner grade ≥ II: a study of 98 patients. Front Endocrinol (Lausanne). (2024) 15:1334414. doi: 10.3389/fendo.2024.1334414 38318295 PMC10841573

[9] Yang Y Li Y Pan Q Bai S Wang H Pan XH. Tibial cortex transverse transport accelerates wound healing via enhanced angiogenesis and immunomodulation. Bone Joint Res. (2022) 11:189–99. doi: 10.1302/2046-3758.114.BJR-2021-0364.R1 PMC9057526 35358393

[10] Buonacera A Stancanelli B Colaci M Malatino L. Neutrophil to lymphocyte ratio: an emerging marker of the relationships between the immune system and diseases. Int J Mol Sci. (2022) 23:3636. doi: 10.3390/ijms23073636 35408994 PMC8998851

[11] Martínez-Urbistondo D Beltrán A Beloqui O Huerta A. The neutrophil–lymphocyte ratio as a marker of systemic endothelial dysfunction in asymptomatic subjects. Nefrologia. (2016) 36:397–403. doi: 10.1016/j.nefro.2015.10.018 26923388

[12] Ong E Farran S Salloum M Gardner S Giovinco N Armstrong DG. Does everything that’s counted count? Value of inflammatory markers for following therapy and predicting outcome in diabetic foot infection. Int J Low Extrem Wounds. (2017) 16:104–7. doi: 10.1177/1534734617700539 28682724

[13] Vatankhah N Jahangiri Y Landry GJ McLafferty RB Alkayed NJ Moneta GL. Predictive value of neutrophil-to-lymphocyte ratio in diabetic wound healing. J Vasc Surg. (2017) 65:478–83. doi: 10.1016/j.jvs.2016.08.108 PMC5336364 27887858

[14] Demirdal T Sen P. The significance of neutrophil-lymphocyte ratio, platelet-lymphocyte ratio and lymphocyte-monocyte ratio in predicting peripheral arterial disease, peripheral neuropathy, osteomyelitis and amputation in diabetic foot infection. Diabetes Res Clin Pract. (2018) 144:118–25. doi: 10.1016/j.diabres.2018.08.009 30176260

[15] Aydın MS Eren MA Uyar N Kankılıç N Karaaslan H Sabuncu T. Relationship between systemic immune inflammation index and amputation in patients with diabetic foot ulcer. J Orthop Sci. (2024) 29:1060–3. doi: 10.1016/j.jos.2023.07.015 37532650

[16] Zhu Y Xu H Wang Y Feng X Liang X Xu L. Risk factor analysis for diabetic foot ulcer-related amputation including Controlling Nutritional Status score and neutrophil-to-lymphocyte ratio. Int Wound J. (2023) 20:4050–60. doi: 10.1111/iwj.v20.10 PMC10681407 37403337

[17] Altay FA Kuzi S Altay M Ateş İ Gürbüz Y Tütüncü EE. Predicting diabetic foot ulcer infection using the neutrophil-to-lymphocyte ratio: a prospective study. J Wound Care. (2019) 28:601–7. doi: 10.12968/jowc.2019.28.9.601 31513494

[18] Serban D Papanas N Dascalu AM Kempler P Raz I Rizvi AA. Significance of neutrophil to lymphocyte ratio (NLR) and platelet lymphocyte ratio (PLR) in diabetic foot ulcer and potential new therapeutic targets. Int J Low Extrem Wounds. (2024), 23:205-16. doi: 10.1177/15347346211057742 34791913

[19] Sun B Chen Y Man Y Fu Y Lin J Chen Z. Clinical value of neutrophil-to-lymphocyte ratio and prognostic nutritional index on prediction of occurrence and development of diabetic foot-induced sepsis. Front Public Health. (2023) 11:1181880. doi: 10.3389/fpubh.2023.1181880 38026334 PMC10630165

[20] American Diabetes Association 2. Classification and diagnosis of diabetes: standards of medical care in diabetes-2019. Diabetes Care. (2019) 42:S13–s28. doi: 10.2337/dc19-S002 30559228

[21] The Chinese Association of Orthopaedic Surgeons (CAOS), Taskforce Group of Tibial Cortex Transverse Transport Technique for the Treatment of Diabetic Foot Ulcers. Expert consensus on the treatment of diabetic foot ulcers using tibial transverse transport (2020). Zhongguo Xiu Fu Chong Jian Wai Ke Za Zhi. (2020) 34:945–50. doi: 10.7507/1002-1892.202003046 PMC8171904 32794660

[22] Edwards J Stapley S. Debridement of diabetic foot ulcers. Cochrane Database Syst Rev. (2010) 2010:Cd003556. doi: 10.1002/14651858.CD003556.pub2 20091547 PMC7144817

[23] Singh AV Varma M Rai M Pratap Singh S Bansod G Laux P. Advancing predictive risk assessment of chemicals via integrating machine learning, computational modeling, and chemical/nano-quantitative structure-activity relationship approaches. Advanced Intelligent Systems. (2024) 6:2300366. doi: 10.1002/aisy.202300366

[24] Singh K Yadav VB Yadav U Nath G Srivastava A Zamboni P. Evaluation of biogenic nanosilver-acticoat for wound healing: A tri-modal in silico, *in vitro* and *in vivo* study. Colloids Surfaces A: Physicochemical Eng Aspects. (2023) 670:131575. doi: 10.1016/j.colsurfa.2023.131575

[25] Liao MM Chen S Cao JR Wang MW Jin ZH Ye J. Early hemodynamics after tibial transverse transport in patients with nonarterial stenosis and arterial stenosis diabetic foot. World J Diabetes. (2023) 14:1784–92. doi: 10.4239/wjd.v14.i12.1784 PMC10784792 38222781

[26] Wang Z Hasan R Firwana B Elraiyah T Tsapas A Prokop L. A systematic review and meta-analysis of tests to predict wound healing in diabetic foot. J Vasc Surg. (2016) 63:29S–36S.e1-2. doi: 10.1016/j.jvs.2015.10.004 26804365

[27] Feng X Ji Y Zhang C Jin T Li J Guo J. CCL6 promotes M2 polarization and inhibits macrophage autophagy by activating PI3-kinase/Akt signalling pathway during skin wound healing. Exp Dermatol. (2022) 32:403–12. doi: 10.1111/exd.14718 36457234

[28] Kim H Wang SY Kwak G Yang Y Kwon IC Kim SH. Exosome-guided phenotypic switch of M1 to M2 macrophages for cutaneous wound healing. Advanced Sci. (2019) 6:1900513. doi: 10.1002/advs.201900513 PMC6794619 31637157

[29] Wang C-S Luo S Jia S Wu W Chang SF Feng S. Balance of macrophage activation by a complex coacervate-based adhesive drug carrier facilitates diabetic wound healing. Antioxidants. (2022) 11:2351. doi: 10.3390/antiox11122351 36552559 PMC9774176

[30] Armstrong DG Lavery LA Harkless LB. Validation of a diabetic wound classification system. The contribution of depth, infection, and ischemia to risk of amputation. Diabetes Care. (1998) 21:855–9. doi: 10.2337/diacare.21.5.855 9589255

[31] van Reijen NS Ponchant K Ubbink DT Koelemay MJW. The prognostic value of the WIfI classification in patients with chronic limb threatening ischaemia: A systematic review and meta-analysis. Eur J Vasc endovascular Surg. (2019) 58:362–71. doi: 10.1016/j.jvs.2019.08.003 31230866

[32] Chen W Chen K Xu Z Hu Y Liu Y Liu W. Neutrophil-to-lymphocyte ratio and platelet-to-lymphocyte ratio predict mortality in patients with diabetic foot ulcers undergoing amputations. Diabetes Metab Syndr Obes. (2021) 14:821–9. doi: 10.2147/DMSO.S284583 PMC7917326 33658817

[33] Fife CE Eckert KA Carter MJ. Publicly reported wound healing rates: the fantasy and the reality. Adv Wound Care (New Rochelle). (2018) 7:77–94. doi: 10.1089/wound.2017.0743 29644145 PMC5833884

